# Where have all the ions gone, long time passing? Tandem quadrupole mass spectrometers with atmospheric pressure ionization sensitivity gains since the mid‐1970s. A perspective

**DOI:** 10.1002/rcm.9354

**Published:** 2022-08-30

**Authors:** Tom Covey

**Affiliations:** ^1^ Sciex Concord ON Canada

## Abstract

The gains in sensitivity since 1975 for quadrupole mass spectrometers equipped with atmospheric pressure ionization (API), and in particular triple quadrupole mass spectrometers (QqQs) since 1981, have been driven by the needs of the environmental, biomedical, agricultural, and other scientific research, industrial, regulatory, legal, and sporting communities to continually achieve lower limits of quantitation and identification. QqQs have realized a one‐million‐fold improvement in sensitivity attempting to address these needs over the past two score years. It is the purpose of this article to describe how that came about, not through an exhaustive review of the literature, but rather by describing what general approaches were used across the industry to improve sensitivity and provide some examples to illustrate its evolution. The majority of the gains came from the ion source and its interface to the vacuum system. “Sampling efficiency” is a measurement of the losses in this area so will be a focus of this review. The discovery of the phenomenon of collisional focusing was key to improving sampling efficiency because it enabled designs that increased the ion‐containing gas loads from the ion source, using staged differential pumping backed by increasingly larger pumps, and prevented the scattering losses of ions in the resulting gas expansion inside vacuum. Likewise, systems with smaller pumps and lower ion‐containing gas loads could be designed with size and cost reduction in mind while maintaining reasonable sampling efficiencies. As a consequence, advancements in the designs of both larger and smaller turbomolecular vacuum pumps were accelerated by pump manufacturers to accommodate the explosive growth in the use of API‐QqQ and API‐ion trap mass spectrometers that occurred in the 1990s and continued into the new millennium. Sampling efficiency was further improved by increasing the ion yield from electrospray by increasing the rate of droplet desolvation. An estimate of the practical limit to further sensitivity improvements beyond what has been achieved to date is provided to shed light on what to expect in the future. Lastly, the implications and unforeseen consequences of the sensitivity gains are considered with a particular focus on how they have enabled a dramatic increase in daily sample throughput on triple quadrupole and other types of mass spectrometers.

## 1 INTRODUCTION

Sensitivity, measured as the ratio of the number of ions detected to the number of molecules injected into the ion source, is a critical metric for many mass spectrometry applications because of its influence on the limit of detection and quantitation (LOD, LOQ), signal‐to‐noise (S/N), dynamic range, mass accuracy, and sample throughput. High sensitivity makes possible the use of additional techniques that improve specificity like ion mobility/mass spectrometry, added levels of tandem mass spectrometry (MS^n^), higher mass resolution, and ion‐chemistry‐based reaction cells. For these reasons, the pursuit of higher sensitivity has been a dominating focus in mass spectrometry since its earliest days. With the advent of atmospheric pressure ionization (API) significant opportunities for improvements to sensitivity emerged.

Applications requiring the accurate and precise measurement of the quantity of targeted components in a sample at low concentrations have consigned the triple quadrupole (QqQ) as the go‐to workhorse instrument for quantitation. This is in part due to the selectivity afforded by tandem mass spectrometry (MS/MS) and in part due to the high ion transmission efficiency that is achieved when operated in the multiple ion monitoring (MRM) mode for targeted analyte detection. Ion losses incurred during the scanning of the quadrupole over a large mass range or the periodic pulsing of the ion beam required for mass analysis with trapping and time‐of‐flight mass (TOF) spectrometers are minimized. Generally speaking, improvements to the sensitivity of atmospheric pressure ionization mass spectrometers were implemented first on the QqQ and would then migrate to other types of mass spectrometers when appropriate.

The sensitivity gains of API‐QqQ systems using electrospray ionization (ESI) have been on the order of one‐million‐fold over the past 40 years and nearly the same gains for the other major method of producing ions at atmosphere, atmospheric pressure chemical ionization (APCI). It is the purpose of this review to explain how this came about by describing the key physical principals utilized to achieve successive gains with examples of how they were implemented by the manufacturers of these systems. An estimate of what sensitivity gains remain to be mined is also provided demonstrating that in practice a limit to improvements is being approached. In addition, examples of opportunities that improvements to sensitivity have provided beyond just lowering limits of quantitation (LOQs) are presented with a particular emphasis on speed of analysis. The enabling of high daily sample throughputs is a direct consequence of these sensitivity gains. This is because sample volumes close to one‐million‐fold less than 40 years ago can now be analyzed while maintaining the same concentration detection limits as earlier. This capability has had a profound ripple effect throughout the entire analytical method and supporting instrumentation by enabling new high‐speed devices for sample dispensing into the mass spectrometer and significantly reducing, in some cases eliminating, sample preparation requirements prior to ionization.

## 2 HISTORICAL OVERVIEW

The development of QqQs^1^ and API^2^, ^3^, ^4^, ^5^, ^6^ occurred at a similar time in the mid‐1970s and early 1980s. Although ionization in vacuum was the predominant approach for creating ions for mass analysis at this time using methods like electron impact and low‐pressure chemical ionization, the first commercial QqQ instrument was dedicated solely to API and launched in March of 1981^7^ for direct analysis of pollutants in ambient air and contraband collected from the surfaces of cargo using APCI.^4^, ^6^, ^7^ Later the same year others entered the QqQ commercial landscape with vacuum‐based electron impact and chemical ionization sources.^7^

**FIGURE 1:**
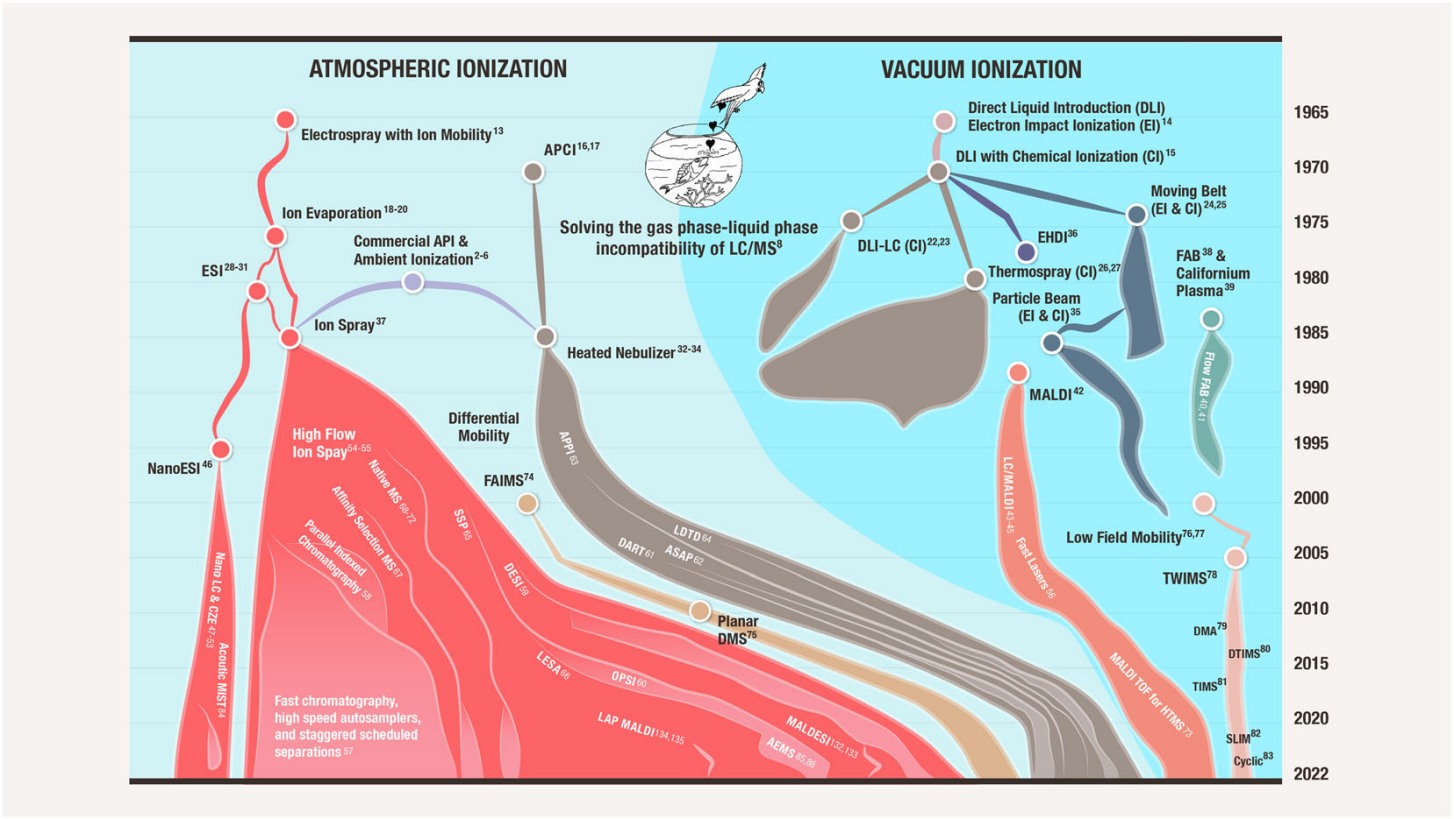
Histomap of the development of LC/MS interfaces from 1967 to present. The emergence of commercial atmospheric pressure ionization (API) LC/MS/MS on triple quadrupole mass spectrometers in the late 1980s laid the foundation for the sensitivity improving technologies to come. The width of the region (x‐axis) that each LC/MS technique occupies approximates its commercial influence relative to the other techniques at that time. The thin line represents pre‐commercialization of the inventions and period of initial publications. The map is bifurcated down the middle into two halves. On the left is shown API LC/MS interfaces that eventually dominated and on the right are those based on ionization under vacuum conditions that ruled in the earlier years. The famous 1980’s bird & fish icon illustrates the incompatibility of the gas‐phase world of MS and liquid‐phase world of LC as viewed at that time, adapted from Arpino.^8^ Recent techniques that multiplex or by‐pass HPLC (ambient ionization) as a means of sample introduction are included. Ion mobility is included in the Histomap even though it is not an LC/MS interface or sample introduction system because of its potential as a high‐speed substitute for some functions of HPLC in emerging systems. LAP MALDI and MALDESI are listed in the electrospray area because they both utilize the ion evaporation ionization mechanism of ESI at atmospheric pressure. Superscripts provide reference to some early publications of the various technologies. This Histomap was adapted from similar Histomaps in the literature^7^, ^9^, ^10^, ^11^ where more detail is provided on the different LC/MS technologies listed. The Histomap concept was originally developed by John Sparks and published by Rand McNally to track the course of world cultural history since the dawn of civilization in a 6‐foot‐long Histomap.^12^ [Color figure can be viewed at wileyonlinelibrary.com]

When it became apparent during the mid‐1980s that introducing liquids into an API source was superior to introducing them into vacuum for ionization, coupling liquid chromatography (LC) to MS with ionization at atmospheric pressure using both APCI and ESI supplanted vacuum ionization as the most widely used approach to interface LC with MS (LC/MS). The Histomap in Figure 1 provides a view of the extent of the switch to API‐MS for LC coupling that occurred in the 1980s and 90s. This Histomap was adapted from similar Histomaps in the literature^7^, ^9^, ^10^, ^11^ where more detail is provided on the different LC/MS technologies listed. Inserted in Figure 1 is the famous bird and fish icon that represented the field^8^ suggesting that merging the seemingly incompatible liquid‐phase world of LC and gas‐phase world of MS was as rift with difficulties as the ill‐advised engagement of Tevye's daughter in the Broadway play “Fiddler on the Roof” when he advised “My dear daughter, a bird may marry a fish. But where would they live?”

The labors of researchers worldwide seeking to solve these problems came to fruition with the emergence of API. This catalyzed a rapid expansion in the use of mass spectrometry where the vast majority of systems deployed were QqQs and three‐dimensional (3‐D) ion traps as dedicated API‐MS/MS systems for coupling to LC. API would migrate to time‐of‐flight (TOF) and Orbitrap high‐resolution mass analyzers around the turn of the century as these instruments improved and moved into the mainstream.

API has both sensitivity advantages and disadvantages compared to the vacuum‐based techniques. APCI is several orders of magnitude more efficient than chemical ionization under vacuum largely because the ionization process is thermodynamically rather than kinetically controlled as it is under vacuum.^2^, ^87^ As a result of improvements to the ESI process, the efficiency of ESI ion production by the Ion Evaporation (IE) mechanism matches that of APCI as it has become generally observed that limits of detection can be similar.

The drawback is that focusing ions created and dispersed at atmospheric pressure is less efficient than if they were created under vacuum where they can be readily focused with electric fields. In a conventional API source using either ESI or APCI where ion production is distributed in a relatively large area and volume of gas, the majority of the ions are lost to the source walls and exhaust ports. This is because at atmospheric pressure gas flows, not electric fields, dominate an ions trajectory. With NanoESI, where ion production occurs immediately in front of the atmosphere‐to‐vacuum aperture, the gas flow created by the vacuum transports the ions with high efficiency. Ions produced in the same region by APCI would have similarly high atmosphere‐to‐vacuum transfer efficiencies as NanoESI but practical limitations have inhibited its general utility for analytical applications.

**FIGURE 2:**
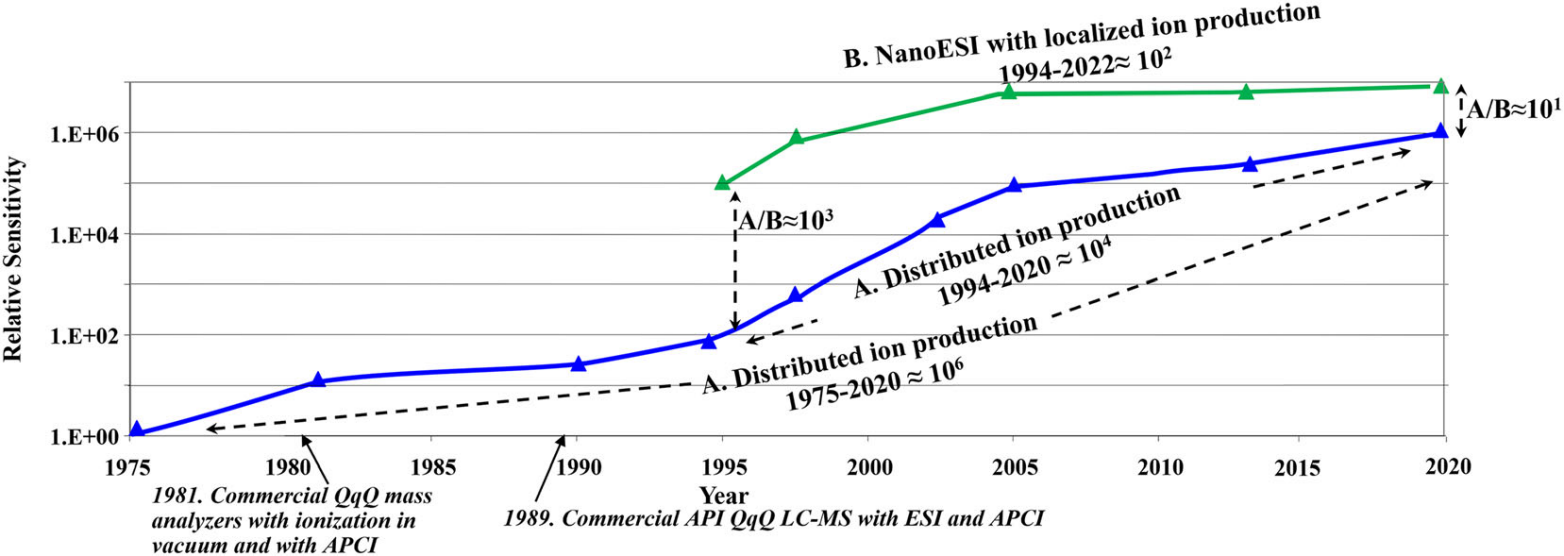
Sensitivity gains of atmospheric pressure ionization quadrupole mass spectrometers since 1975. A, Trace tracking the sensitivity gains for conventional API sources that produce ions widely distributed throughout the ion source. This includes APCI air monitoring, APCI thermal desorption, other forms of ambient sampling (ambient ionization), and the introduction of samples in fluids for APCI and ESI at liquid flow rates relevant to conventional HPLC (~50–1000 μL/min). An improvement of ~1,000,000‐fold has occurred since the beginning and ~10,000‐fold since 1994. B, Trace tracking the sensitivity gains for NanoESI (<1 μL/min) where ion production is localized within the vacuum drag region immediately in front of the atmosphere‐to‐vacuum aperture. An improvement of ~100‐fold in sensitivity has occurred since its introduction in 1994. The data for these graphs was taken from the records of the first manufacturer of dedicated API‐QqQ instruments. Similar graphs could be produced by other manufacturers albeit not going as far back in time. Adapted from Thomson^88^ [Color figure can be viewed at wileyonlinelibrary.com]

Figure 2A shows the million‐fold gain that occurred since the mid‐1970s with conventional API sources originally using APCI thermal desorption techniques for air pollution tracking and cargo contraband detection. Liquid introduction with both ESI and APCI would follow for conventional chromatography LC/MS applications and much later other methods of ambient sample introduction (ambient ionization). The data in this graph comes from the records of the first manufacturer of dedicated API‐QqQs^88^ and similar graphs could be constructed by other manufacturers of API‐MS although not going as far back in time.

Figure 2B is the special case of NanoESI developed in 1994^46^ where ionization at atmospheric pressure is localized in the vacuum drag region and transport is highly efficient. The improvements shown in Figure 2 rival the gains predicted by Moore's Law for transistor density in microelectronic chips that states it will double approximately every 2 years. The discoveries underpinning the sensitivity gains, and some examples of the devices employed to achieve them, are described in the following sections to illustrate the extent of the efforts deployed across the industry.

## 3 SENSITIVITY AND SAMPLING EFFICIENCY

### 3.1 Background

Advances in electrospray ionization efficiency, the efficiency of transporting ions from atmosphere‐to‐vacuum, the confinement of the ion beam in the gas expansion upon entering vacuum, and the confinement of the ion beam in the collision cell have been the major contributors to the improvements in the sensitivity of API‐QqQ instruments and account for the gains indicated in Figure 2. Details as to how these four efficiencies have been improved have been reviewed elsewhere^9^, ^89^ but general points will be made about where the losses are the greatest, the most gains have been made, how that was achieved, and what is left to be mined in the future. This is best understood by considering separately each of the components of the *sampling efficiency*.

### 3.2 Sampling efficiency, a subset of the sensitivity metric

Sampling efficiency is the percentage of molecules introduced into the ion source that arrive at the entrance of the mass analyzer. It is measured by recording the signal at the detector from a known concentration of a molecule (sensitivity measurement) then accounts for the losses from the mass analyzers and collision cell which have been previously determined from ion current measurements after each of these elements.^90^, ^91^, ^92^ Sampling efficiency quantifies collectively the losses from three processes: (1) the efficiency of ionization in the ion source, (2) the efficiency of transporting ions from atmosphere to vacuum, and (3) the efficiency of confining the ion beam as the gas initially expands in the vacuum.^90^, ^91^, ^92^ A sampling efficiency of 100% means all analyte molecules are ionized at atmosphere, transported into vacuum, and collimated into a well‐defined ion beam without scattering losses in the ion optics prior to mass analysis. It is the theoretical maximum that can be obtained from an API source.^91^, ^92^ Since it does not account for losses during mass filtering or collisionally activated dissociation (CAD) fragmentation in the collision cell,^93^ which are included in the sensitivity metric, it is independent of the type of mass analyzer which can vary widely with regards to their inherent ion transmission and duty cycle characteristics. Neither sampling efficiency nor sensitivity consider chemical noise levels which constitute the denominator of signal‐to‐noise (S/N) calculation used for determining LOQs.

**FIGURE 3:**
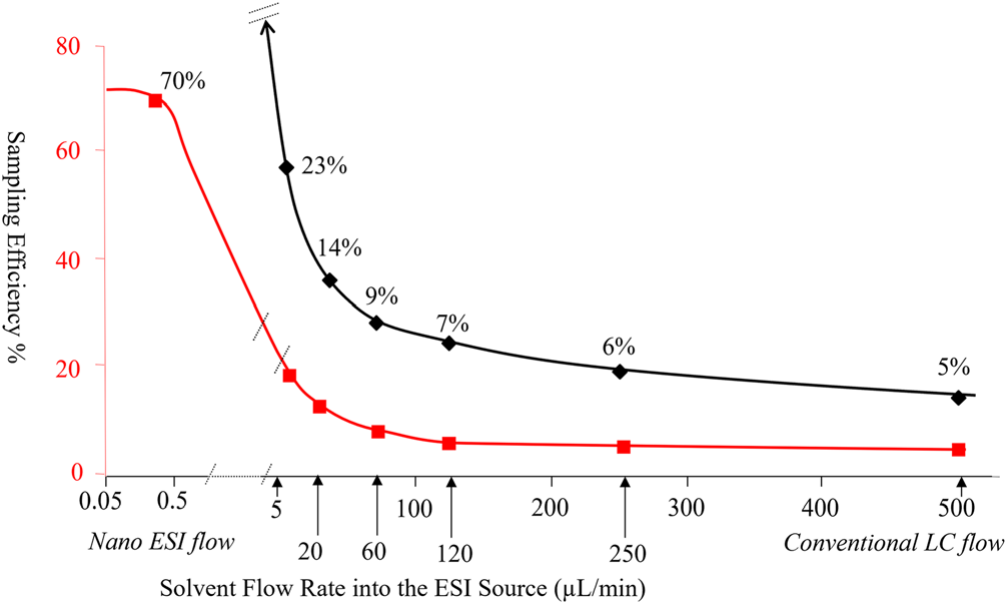
Ion sampling efficiency of reserpine with electrospray ionization at inlet fluid flows spanning NanoESI to conventional HPLC flow rates in 2020. The black graph is an expansion of a portion of the red graph to illustrate the trends more clearly in the high and microflow regimes. A sampling efficiency of 100% means all molecules of analyte injected into the ion source enter the mass analyzer as ions. NanoESI is shown to be near the theoretical maximum. Sampling efficiency is measured by recording the signal at the detector from a known concentration of a molecule.^90^, ^91^, ^92^ The previously measured losses from the mass analyzer and collision cell are accounted for to establish the number of ions arriving to the entrance of the mass analyzer. Graph adapted from Covey^7^ using data from Schneider et al^92^ from a contemporary triple quadrupole mass spectrometer (API 7500) designed for high sampling efficiency [Color figure can be viewed at wileyonlinelibrary.com]

In Figure 3 NanoESI is shown to be today near the theoretical maximum sampling efficiency with the test compound reserpine. As seen in Figure 2B the sensitivity of NanoESI has risen ~100‐fold since its inception in 1994. Sampling efficiency has improved since then by ~10‐fold and with the additional improvements of ~10‐fold to collision cell focusing a 100‐fold increase in sensitivity was achieved.

Figure 3 shows the sampling efficiency for conventional API, which includes ESI and APCI operating at conventional liquid flow rates, has reached ~ 5% for this compound, an improvement of ~10,000‐fold since 1994,^92^ as displayed in Figure 2A. Figures 2A and 2B illustrate that the sensitivity of conventional API and NanoESI have been converging over time and are now within an ~10‐fold difference compared with a 1000‐fold difference in 1994, as will be further discussed in the next sections. If one considers that the sample capacity difference between a conventional HPLC 2.1 mm i.d. column vs a 0.1 mm i.d. column used for NanoESI is ~400‐fold, it is now the case that lower concentration limits of quantitation (nM, ng/mL, ppb, for example) can be achieved with high flow systems in situations where available sample for analysis is not severely limited, as is the case with many pharmaceutical drug discovery and development applications. In cases where it is limited, such as the analysis of the components of single cells or extracts from 2‐D gel electrophoresis, NanoESI still has the advantage.^92^

### 3.3 Ionization efficiency

Ionization efficiency is the component of sampling efficiency measuring the percentage of molecules introduced into the ion source that become gas‐phase ions or small clusters with solvent at atmospheric pressure. The improvements to ESI efficiency have primarily involved increasing the rate of reducing the diameters of charged droplets to their Rayleigh limit. Upon reaching this limit coulomb explosions complete their final droplet size reduction to ion emission diameters of ~10 nm within a few tens of microseconds.^20^, ^94^, ^95^ The strong electric field at the surface emanating from the excess charge within these small droplets launches the bare ion or ion bound to a few solvent molecules (cluster ions) by field desorption to the gas phase. This is the basic tenant of the Ion Evaporation Theory of Iribarne and Thomson.^18^, ^19^, ^20^, ^21^, ^94^, ^95^, ^96^

**FIGURE 4:**
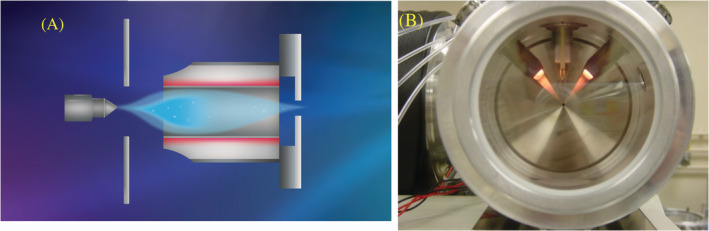
Improvements to ionization efficiency by increasing charged droplet desolvation rate through the use of heated chambers or by the injection of hot auxiliary gasses. (A), Heated desolvation chamber for NanoESI of dimensions 2 mm i.d. × 1 cm long. The gas flow through it is generated by the MS vacuum because it is sealed to the atmosphere‐to‐vacuum interface plate. Heated ion pipes serve the same function. Adapted in part from Schneider et al.^91^ (B), High‐temperature gas injectors (800°C) for high flow rate ESI.^9^, ^55^ The gas nebulized spray (~1 L/min) achieves sonic velocities a few mm from the nozzle at which point it rapidly breaks. Gas from the immediate surroundings is entrained into the spray in this region at a volumetric ratio of ~10/1 expanding its volume. The mutual self‐repulsion of the charged droplets further increases their scatter. When the entrainment gas is injected into this region (~10 L/min) at high temperatures rapid droplet size reduction occurs. The evaporative cooling effect prevents the droplets from exceeding 50°C. Ions are emitted from the droplets in a cooler region avoiding thermal degradation. Reproduced in part from previous studies^7^, ^9^, ^55^ [Color figure can be viewed at wileyonlinelibrary.com]

NanoESI conditions involve using liquid flow rates at below 1 μL/min which allows the ESI emitter to be positioned close to the atmospheric aperture where the vacuum drag insures that nearly all of the droplets are drawn into the mass spectrometer. The charged droplets have diameters distributed around ~1 μm as generated by a NanoESI emitter. To reach the Rayleigh limit of ~100 nm, additional size reduction is required.^94^, ^95^, ^96^ The residence time in the vacuum drag region is on the order of tens of microseconds before severe cooling occurs in the free jet expansion into vacuum. Passing the charged droplets through appropriately heated chambers or tubes in the vacuum drag region results in a 2–3‐fold improvement in ion production^91^ because a greater percentage of them are evaporated to the size where coulomb explosions are initiated. An example of a heated chamber for NanoESI is shown in Figure 4A. Under these conditions the ionization efficiency of NanoESI approaches nearly 100% for molecules like reserpine with high surface activity and are fully ionized in solution, so called “electrospores”.

The ionization process of ESI is generally considered to be most accurately described by the Ion Evaporation Model which originated during the study of the formation of ions from rain clouds^19^ then was applied to mass spectrometry applications.^20^, ^21^ The analytical derivations in that work made it obvious that charged droplet and ion production from large volumes of electrically charged liquids could be enhanced by processes that contributed to droplet size reduction such as through the use of additional shear forces.^19^, ^20^ Pneumatic nebulizers were introduced in the early 1980s to create droplets from fluid flows that were too great, in the tens to hundreds of μL/min, for electric fields alone to disperse as well as to increase the rate of desolvation to the Rayleigh limit.^21^, ^37^ The Ion Spray^37^ interface emerged from this effort, so‐called because it combined elements of Thomson's Ion Evaporation and Fenn's Electrospray interfaces, thus the name "Ion Spray".^55^ As depicted in Figure 1, Ion Spray contributed to the expansion in the use of ESI by making it compatible with conventional chromatography. Improvements to this approach provided some sensitivity gains by further increasing desolvation rates with gentle heat^54^, ^97^ but the ionization efficiency remained significantly lower than APCI where the ionization efficiency is understood to be close to 100% given the gas‐phase acidity or basicity of the analyte is greater than the reagent gas.^2^, ^5^, ^87^, ^98^

When APCI is used for LC coupling the vaporized solvent is ionized with an electrical discharge or other means which then serves as a reagent gas to ionize the analytes present at a much lower gas‐phase concentration. APCI‐based LC interfaces vaporize both the solvent and dissolved analytes to gas‐phase entities before ionization at flow rates approaching 1 mL/min, as demonstrated by Carroll, Henion, and Thomson.^17^, ^32^, ^33^, ^34^ The discrepancy between the sensitivity of APCI and ESI at these flows made it apparent that improvements could be made to high‐flow ESI with a more careful consideration of the heat transfer process. The pneumatically generated charged droplets from Ion Spray average ~10 μm in diameter when generated at these flow rates and can be evaporated to their Rayleigh limits within their flight time in the ion source (~1 ms) if they are deeply entrained in gas at temperatures as high as 800°C.^55^

Figure 4B is a photograph of an ion source that achieves this condition. Temperatures of this magnitude seem alarming considering the possibility of thermal degradation. The temperature of the liquid, however, in the evaporating droplet reaches a maximum of only ~50°C due to the evaporative cooling effect^99^ and only increases when the droplet is reduced to a dry residue at which time it would spontaneously ignite if it remained at this temperature. The nebulizer gas velocity is sonic at the nozzle and rapidly decreases causing an uptake or entrainment of surrounding air as dictated by the laws of conservation of momentum. The volumetric flow rate of the entrained gas (~10 L/min) is approximately 10× the flow rate of the nebulizer gas (~1 L/min). When the entrainment gas is injected at a high temperature in the region where the nebulizer gas velocity is reducing, heat transfer and desolvation to the Rayleigh limit occur rapidly. As the droplets approach the ion emission diameter of 10 nm they enter a cooler region of the ion source near the vacuum entrance where ions clustered with solvent molecules, that absorb additional vibrational energy from the surrounding gas, are emitted from the droplet.^55^ The residence time of the ion‐solvent cluster in this region is a few tens of microseconds as it is swept out by the vacuum drag of the mass spectrometer. Thus, thermal degradation is primarily avoided and declustering is achieved by collisions with background gas in vacuum in a controlled fashion with a direct current (DC) potential and the use of a dry counter‐current flow of nitrogen sometimes referred to as a curtain gas.^3^

This development in 2001 provided ionization efficiency gains 20‐ to 30‐fold above the previous generation of ESI sources^9^, ^55^ at high fluid flow rates bringing the sensitivity of high flow rate ESI at par with APCI. As stated earlier, APCI is understood to have an ionization efficiency, under favorable gas‐phase ion‐molecule reaction conditions, approaching 100%. The ionization efficiency of ESI is close to 100% only with electrospores. The physical–chemical properties of the analyte and its surrounding liquid medium determine the absolute ionization efficiency with ESI. The situation remains for both nano and high flow modes of operation that the absolute ionization efficiency can vary by three orders of magnitude across chemical space.^100^ The primary means employed to improve this is by altering the physical–chemical properties of the analytes by derivatization to improve their surface activity and their ability to be charged in solution or by the addition of another charged ion such as ammonium, sodium, lithium, formate, acetate, or fluoride (ammonium fluoride) to the solvent to form charged adducts^101^, ^102^, ^103^, ^104^, ^105^, ^106^ or enhance the ionization process by improving the surface activity. A 1000‐fold improvement in the ionization efficiency for poor ionizers using derivatization has been demonstrated.^7^

### 3.4 Transport Efficiency

Improvements to the transport of ions from atmosphere‐to‐vacuum have been primarily achieved by increasing the amount of ion‐containing atmospheric gas drawn into the vacuum system. This has been done by reducing the resistance to flow of the atmospheric aperture by increasing its diameter or, in the case of ion pipes, shortening the length of the channel or increasing its diameter. This requires increasing the vacuum pumping speed to accommodate the increased gas loads. Apertures in thin plates, defined as having a length‐to‐diameter ratio (L/D) of ~1, in the mid‐1970s had diameters of 0.025 mm (0.0005 mm^2^)^2^ and increased to ~1.5 mm. (1.8 mm^2^) in recent times.^92^, ^107^ This has led to an increase in the ion‐containing gas being drawn into the vacuum entrance optics by over 3600‐fold. The diameters and lengths of ion pipes, defined as having an L/D of >20, have also been adjusted to achieve gains in gas throughput.^9^, ^108^, ^109^

**FIGURE 5:**
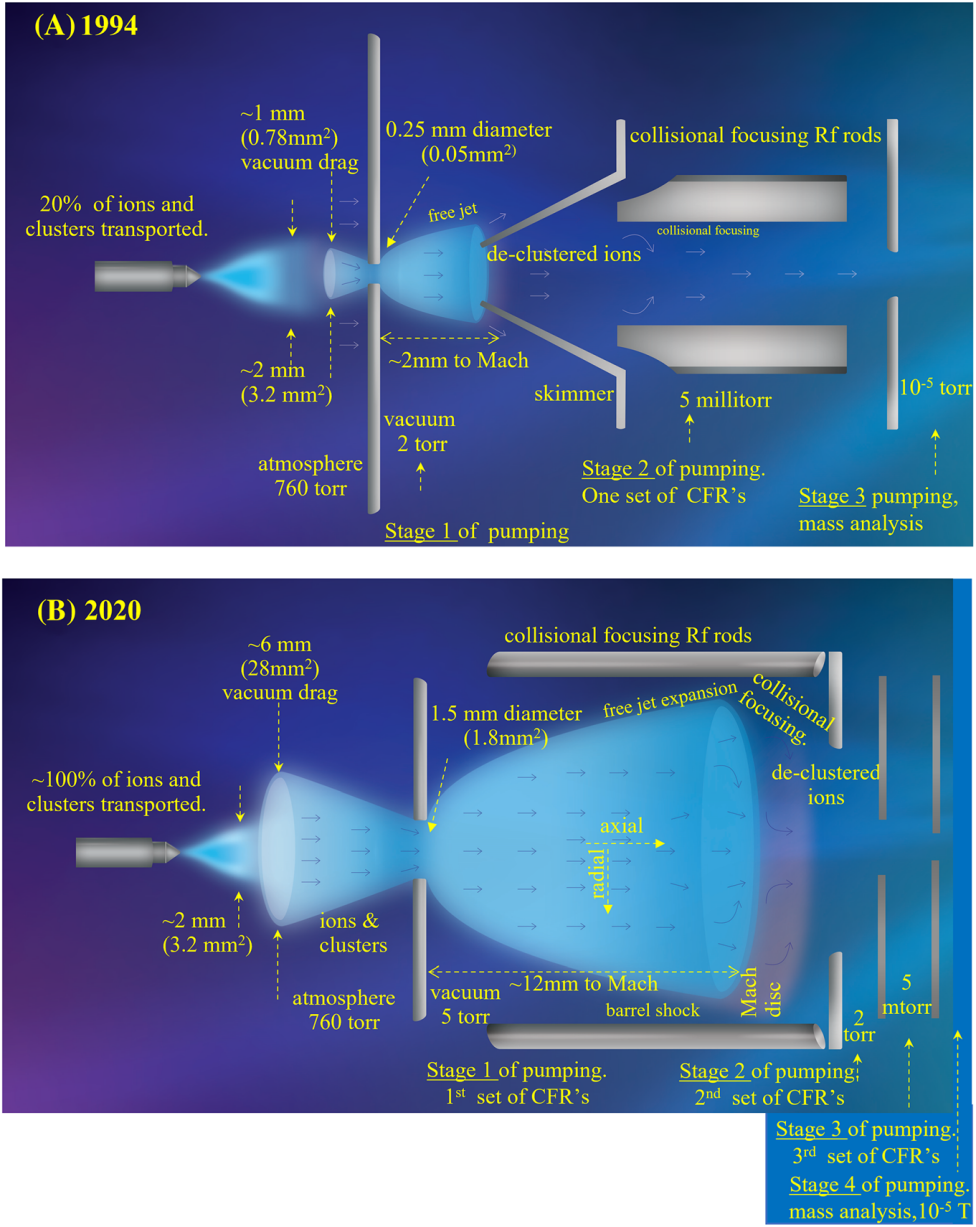
Drawings of a NanoESI sprayer on API interfaces representative of those used in 1994 and 2020. The ESI spray plume where ions are created, the vacuum drag region in front of the vacuum aperture, and the free jet gas expansion into vacuum are drawn ~ to scale relative to the aperture diameter in a thin plate with pressure drops as indicated. The diameter of the vacuum drag region is calculated to be ~4× the aperture diameter in a thin plate and extends from the plate the same distance.^9^ This region can be mapped by rastering a NanoESI needle in three dimensions around the atmosphere‐to‐vacuum aperture and recording the signal. The distance from the aperture to the Mach disc, X_M_, is given by:X_M_ = 0.67 D_0_ (P_0_/P_1_)^1/2^where D_0_ is the aperture diameter as indicated, P_0_ is the atmospheric pressure (760 Torr), and P_1_ is the downstream pressure in the vacuum chamber as indicated. (A), **1994**. Vacuum aperture = 0.25 mm. Approximately 25% of the ion‐containing spray plume is drawn in by the vacuum drag region based on the ratio of the area of the spray, where ion production is greatest, to the area of the vacuum drag region (0.78mm^2^/3.20 mm^2^ = 24.4%). The free jet is sampled with a passive skimmer (0.75 mm diameter) showing some ion losses during transfer. CFR = collisional focusing rods with radiofrequency (RF)‐only fields used post skimmer to focus the remaining ions at 5 mTorr prior to mass analysis at 10^−5^ Torr. This represents three stages of differential pumping using collisional focusing in one stage. (B), **2020**. Vacuum aperture = 1.5 mm. 1994. 100% of the ion‐containing spray plume is drawn in by the vacuum drag region based on the ratio of the area of the spray, where ion production is greatest, to the area of the vacuum drag region (3.2 mm^2^/28 mm^2^ > 100%). The free jet has expanded in both distance (12 mm) and diameter (12 mm) from the aperture increasing ion scattering losses from collisions unless collisional focusing elements are used in this area immediately downstream of the aperture as shown. This represents four stages of differential pumping using collisional focusing in three stages. Since 1994 the transport efficiency has improved 3–4‐fold and with the addition of heated regions (2–3‐fold) the sampling efficiency has improved ~10‐fold. With the addition of the improvements to collision cell efficiency (10‐fold) the sensitivity improvement from 1994 to 2020 for NanoESI is ~1 × 10^2^ (Figure 2). [Color figure can be viewed at wileyonlinelibrary.com]

A situation on a typical instrument in 1994, when NanoESI was first reported,^46^ is depicted in Figure 5A. A vacuum aperture diameter in a thin plate of 0.25 mm was typical at that time and heating of the spray plume was not implemented effectively at the beginning. The sampling efficiency is 25% in this situation when the spray is heated^91^ and 10% when it is not. A typical NanoESI plume can be observed to have a diameter of ~2 mm and encompass an area of ~3.2 mm^2^ in cross section. The vacuum drag region, that is the area of the API source evacuated by the MS vacuum, encompasses an area of 0.78 mm^2^. The ratio of these areas (0.78/3.2 = 0.24) estimates the efficiency of transporting the ions created by a NanoESI emitter into vacuum. The good agreement of the sampling efficiency measurement (>70%, Figure 3) and the observed percentage of the spray transported is evidence that the ionization efficiency is approaching 100% in the heated spray case.

A situation in 2020 is depicted in Figure 5B. With a vacuum aperture diameter of 1.5 mm, the vacuum drag area is 28 mm^2^ in cross section evacuating a region approximately 6‐fold greater than the NanoESI spray inhaling all of it. Both ionization and transport efficiencies are approaching the theoretical maximum as indicated by the measured sampling efficiency of >70%^92^ (Figure 3). Nearly all molecules are ionized, transported to vacuum, and focused in the entrance optics prior to mass analysis.

The improvement to NanoESI sampling efficiency since its inception has been ~ 10‐fold as a result of the increase in the aperture area relative to the spray area to improve transport (3–4‐fold) and the use of heated desolvation regions (2–3‐fold) to improve ionization efficiency. Considering the additional 10‐fold gain in ion transmission from improved collision cells (further discussed in the section on *Focusing Efficiency*) the ~100‐fold NanoESI sensitivity gain shown in Figure 2 can be understood. The reason it is not greater than this is because the sampling efficiency was very high at its inception leaving less room for improvement over time.

**FIGURE 6:**
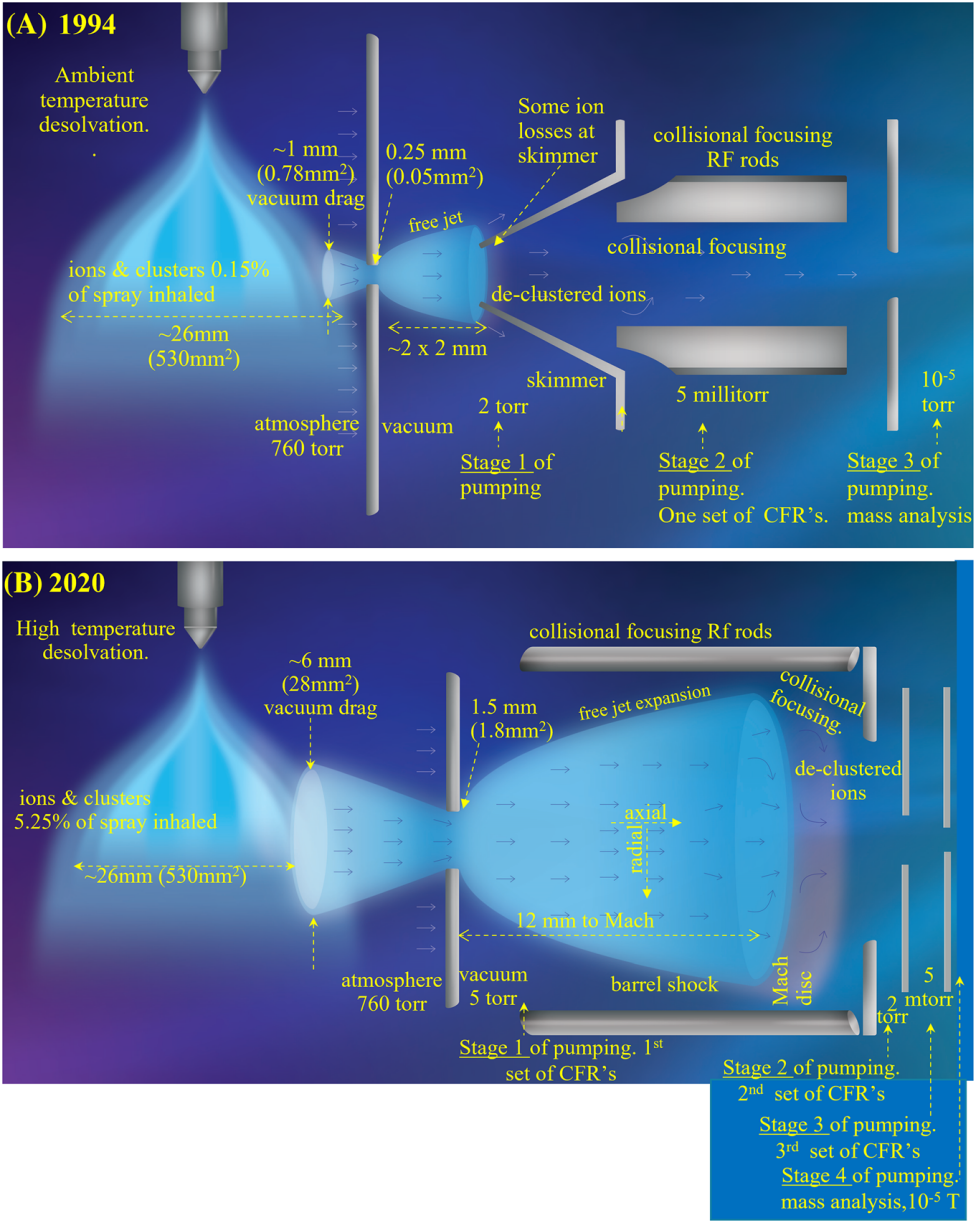
Drawing of a gas nebulized high fluid flow ESI sprayer (Ion Spray) on API interfaces representative of those used in 1994 and 2020. The same API interfaces and entrance optics as in Figure 5. The vacuum drag region in front of the vacuum aperture and the free jet gas expansion into vacuum are drawn ~ to scale relative to the aperture diameter in a thin plate with pressure drops as indicated. The vacuum drag region extends from the plate a distance ≈ its maximum diameter which is ~4× the aperture diameter. The ESI spray plume is drawn at ~ 50% of its diameter relative to the above‐mentioned components to conserve figure space. CFR = collisional focusing rods with RF‐only fields. (A), 1994. Approximately 0.15% of the ion‐containing spray plume is drawn in by the vacuum drag region based on the ratio of the area of the spray, where ion production is greatest, to the area of the vacuum drag region (0.78 mm^2^/530 mm^2^ = 0.15%). (B), 2020. Approximately 5.25% of the ion‐containing spray plume is drawn in by the vacuum drag region based on the ratio of the area of the spray, where ion production is greatest, to the area of the vacuum drag region (28 mm^2^/530 mm^2^ = 5.3%). Vacuum drag region extends from the plate a distance ≈ its maximum diameter. The transport efficiency has improved 36‐fold as determined by the aperture area increase (1.8 mm^2^/.05 mm^2^) and improved ion focusing in vacuum. With the addition of the improvements to ionization efficiency (~25‐fold) and improvements to collision cell efficiency (10‐fold) the sensitivity improvement from 1994–2020 for sources where the ionization is distributed in a large region is ~1 × 10^4^. [Color figure can be viewed at wileyonlinelibrary.com]

The gains in sampling efficiency have been greater for modes of ionization where ion production is widely distributed in the source because there has been more room for improvement. Figures 6A and 6B compare the situation in 1994 and 2020 for fluid flow rates in the 10 to 1000 μL/min range. The areas of the vacuum drag regions are the same as in the NanoESI situations in Figure 5 (0.78 and 28 mm^2^) but the area over which the ions are produced is much larger. Both the nebulizer gas expansion and the space charge repulsion of the large, charged droplet population produce a spray plume that has a diameter of approximately 26 mm (530 mm^2^) in the region where optimal signal is empirically achieved. Based on the ratios of the observed spray cross‐sectional area and the calculated cross‐sectional area of the vacuum drag region in 2020 approximately 5.2% (28 mm^2^/530 mm^2^) of the ion‐producing spray can be transported. Similar to the case with NanoESI, the measured sampling efficiency of 5.25%^92^ in 2020 (Figure 3) closely matches the estimated transport efficiencies based on the ratio of the spray to vacuum drag cross‐sectional areas. This indicates that, as it did with NanoESI, the efficiency of ion production, in the portion of the spray being evacuated by the vacuum drag, must be close to 100% with the high‐temperature desolvation conditions used. However, approximately 95% of the ions and cluster ions produced are lost to the walls and exhaust ports of the ion source because they are scattered outside of the vacuum drag region by the gas flows and droplet space charge.^9^, ^55^

Figures 6A and 6B show that the increase in the aperture areas, accompanied by an increase in pumping speed, from 0.05 mm^2^ (0.25 mm diameter) in the mid‐1990s to 1.8 mm^2^ (1.5 mm diameter) in the 2020s resulted in a 36‐fold increase in the area sampled and volume of ion‐containing gas drawn from the ion source during this time. The sampling area in the 1970s of 0.0005 mm^2^ (0.025 mm diameter) has increased 3600‐fold. With the efficiency of ion production increasing 20–30‐fold in this flow range using more effective desolvation in the early 2000s, the increase in the sampling efficiency has approached 1000‐fold since 1994 and 100,000‐fold since the 1970s. Including the additional 10× improvement to collisions cells, the overall sensitivity gain has been ~ 10,000‐ and ~ 1,000,000‐fold since 1994 and 1975, respectively.

The losses incurred when ions are distributed throughout a large volume of an API source immediately suggest that focusing and concentrating the ions at atmospheric pressure near the orifice should improve the transport efficiency. However, at the most basic level, it can be shown that any attempt to increase the concentration of ions using DC focusing fields at atmospheric pressure, where ion motion is governed by ion mobility, is frustrated by Maxwell's equations (specifically Gauss' Law). ^9^ This explains the lack of success in doing so. Flat and concentric funnel‐shaped high‐voltage DC lenses have been studied showing at best 2× gains under controlled conditions^110^, ^111^ that are erased by the presence of even moderate gas flows from nebulizers, source exhausts, and aperture curtain or counter flow gases. The forces exerted by gas flows under typical operating conditions and the space charge created by large populations of charged droplets on an ion's fate at atmospheric pressure dominate.^9^

RF ion guides have been constructed to take advantage of the collisional focusing principle at atmospheric pressure.^112^ Collisional focusing has been successful at lower pressures in the Torr to milliTorr pressure regime and has played a key role in the sampling efficiency gains as further discussed in the next section. While the same technique can in principle be applied at atmospheric pressure, the focusing effect is slow (in time) at high pressure, so either a small device is required (which makes it unsuitable for collecting and focusing ions from a large‐volume source) or a very long device is required to give the ions sufficient time to focus to the center. RF fields have demonstrated the ability to guide ions from a NanoSpray needle positioned outside the vacuum drag region (~10 cm) without losses. Also demonstrated was a 10‐fold improvement in sensitivity versus a NanoESI emitter close to the vacuum entrance of the experimental system used indicating a focusing effect.^112^ However, this could not improve NanoESI sensitivity on a large aperture instrument as described here because efficiencies are approaching 100%. Sensitivity improvements with this approach for sample introduction liquid flow rates above the nanoflow regime or with sources where the ions are otherwise distributed throughout the source volume have yet to be demonstrated. It is to be expected that extraneous gas flows and droplet space charge will disrupt the ion beam formed in the potential well of these devices.

Improvements in concentrating ions by manipulating gas flows instead of electric fields have painted a similar story. Other forms of nebulization that avoid the use of high‐velocity gases, such as ultrasonics, demonstrated limited to no benefit for high flow rate operation^113^ most likely because of the droplet space charge issue. Gas flow focusing using gas pressure amplifiers^114^ and swirling vorticular flows^115^ have also been tried to reduce the spray dispersion. In some instances, as shown with the RF‐focusing devices described above, they have effectively transported ions from NanoESI emitters over distances of ~10 cm to the vacuum aperture with minimal losses. But they have not shown an improvement in sensitivity under typical operating conditions for either nano or high flow rate sample introduction.

Improving upon today's 5% sampling efficiency for distributed ion production to bring it closer to the theoretical maximum achieved by NanoESI would require a factor of approximately 10‐fold improvement and is the topic of research worldwide but the way forward is not clear other than to increase the aperture size and draw more ion‐containing gas from the API source. The latter approach, however, presents additional complications discussed in the next section.

### 3.5 Ion beam focusing efficiency in vacuum

Ion beam focusing efficiency in vacuum is the component of sampling efficiency that describes the effectiveness of preventing ion scattering losses as they enter the vacuum system in a rapidly expanding gas. Increasing the transport of ions into vacuum by increasing the aperture and pumping speed will be to no avail unless steps are taken to reduce the scattering losses that become more severe as the aperture diameters are increased and the gas expansion becomes more violent.

**FIGURE 7:**
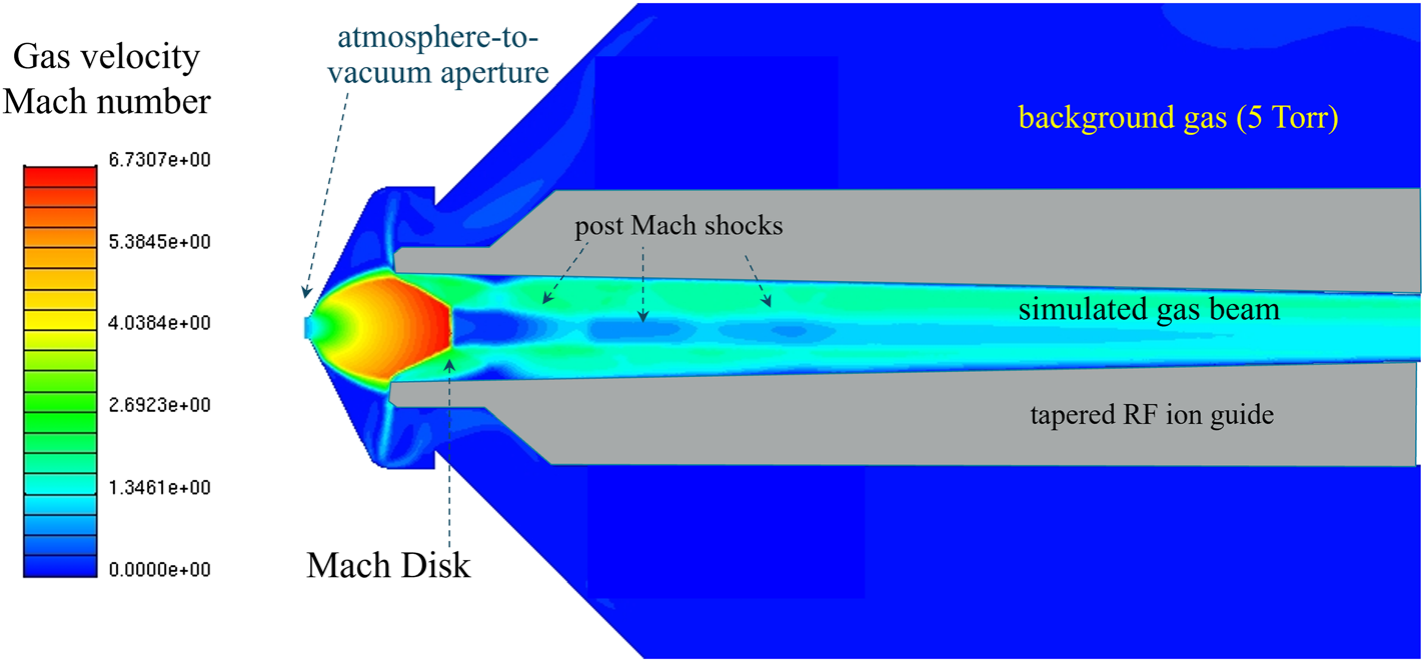
Computational fluid dynamics (CFD) model of the gas flows and velocities during expansion into vacuum within a set of RF‐only rods. Beyond the initial Mach disc additional shock waves develop in the gas beam that will scatter the ions unless they are confined with collisional focusing. In this case the RF ion guide is a tapered dodecapole. Figure adapted in part with permission from Javaheri and Schneider^118^ [Color figure can be viewed at wileyonlinelibrary.com]

A well‐defined supersonic gas expansion occurs downstream of the aperture with ions and gas accelerated to Mach velocities in a jet structure that has been thoroughly studied and characterized in the field of rarified gas dynamics.^116^, ^117^ Figures 5, 6, and 7 show the basic structure of the barrel‐shaped expanding jet terminating in a shock wave, referred to as the Mach disc, where the supersonic gas velocity becomes sub‐sonic through a thin re‐compression region. Inside the barrel shock, the silent zone, ions and molecules move at an equal speed in the same direction and undergo strong and rapid adiabatic cooling. The gas molecules with entrained ions follow straight streamlines inside the barrel shock while beyond the Mach disc the motion of the ions and gas becomes random as indicated by the arrows in Figures 5 and 6. In the transition between directed and random motion ions are lost in the resulting shock wave where ions and gas molecules undergo many collisions and are scattered. Beyond the Mach disc additional satellite shock waves will propagate as shown in the gas velocity flow simulation of Figure 7 where further losses occur by the same process.

**FIGURE 8:**
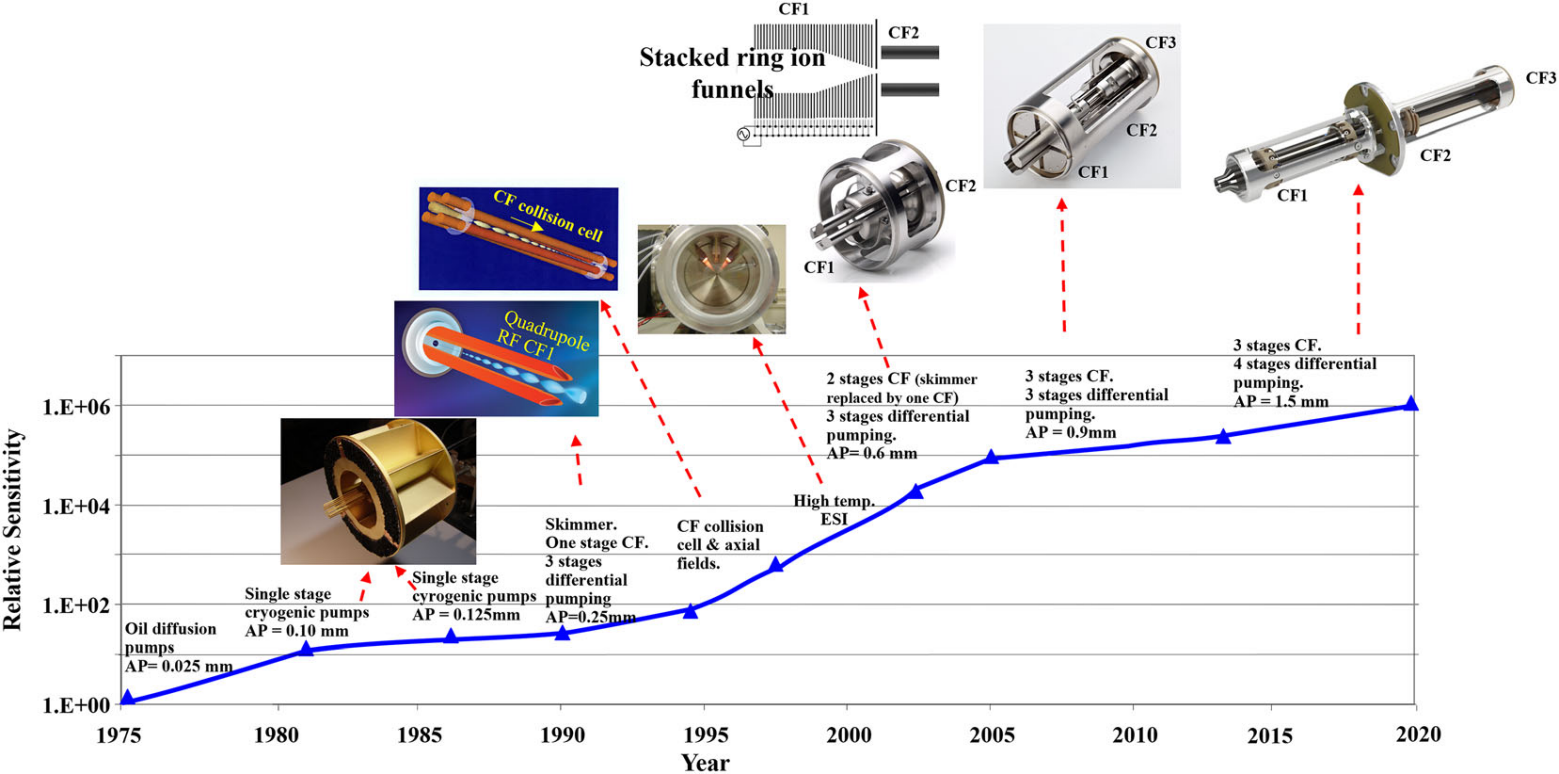
Same graph as Figure 2B with examples of technologies used to achieve incremental sensitivity gains for API‐MS since 1975. Transport efficiency gains were achieved by increasing the aperture diameter (AP), increasing the pumping speed, and utilizing the principle of collisional focusing with different types of RF lens geometries consisting of multipoles or stacked lens arrays to focus the ions through the high‐pressure regions. Collisional focusing (CF) enabled the use of turbomolecular pumps of increasing size (>500 L/s) and differential pumping to achieve greater ion throughputs. In the early stages of development between 1975 and 1990 oil diffusion pumps were used followed by high‐speed single‐stage cryogenic pumps (up to 100,000 L/s) without collisional focusing [Color figure can be viewed at wileyonlinelibrary.com]

To minimize these losses the development of specialized optics to contain the ions has been required. It was the discovery of collisional focusing in 1992^119^, ^120^ that gave birth over the years to a variety of high‐pressure RF‐based ion guides having various quadrupole, multipole, and lens arrays shaped as funnels^121^ that could operate in the milliTorr to Torr pressure regime and bring these scattering losses under control. These collisional focusing devices have continued to be improved upon over the years.^107^, ^118^ Examples of some of them based on both RF‐only rod and lens arrays are shown in Figure 8 illustrating the gradual increase in the number of differentially pumped stages each containing collisional focusing elements of different geometries.

**FIGURE 9:**
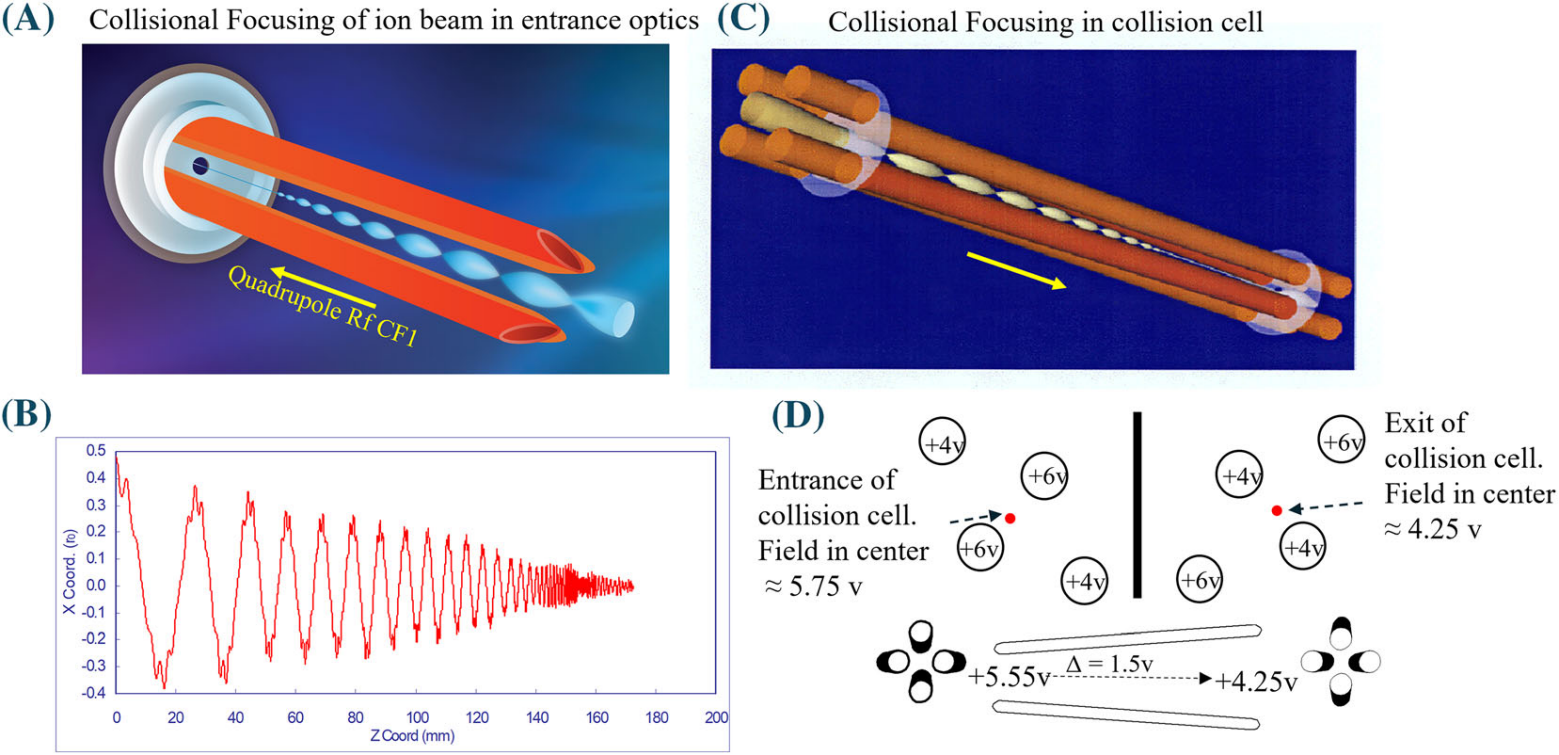
Collisional focusing principle. (A), Artist's rendition of quadrupole rods at 5 mTorr focusing the ion beam before entering the mass analyzer at 10^−5^ Torr. The ion beam passes through a small aperture while the gas is restricted by the lens. (B), Simulation of the collisional focusing effect. The radial energy obtained from the RF field toward the rods is dampened by the collisions with background gas molecules allowing the beam to collapse into the pseudopotential well on the center line. (C), Artist's rendition of a collision cell at 5 mTorr showing the same focusing effect and efficient transport of product ions though the small exit aperture. (D), The collision cell rods are tipped to create an axial field gradient along the length of the rods. Since the collisions deplete the axial kinetic energy of the ions they will stop unless pushed through the cell by an external force. This eliminated so‐called cross‐talk between different MRM channels that had common product ions when they were rapidly switched. This approach to creating an axial field gradient is referred to as a LINAC.^122^, ^123^ There are a variety of other means to create an axial field gradient to serve the same purpose [Color figure can be viewed at wileyonlinelibrary.com]

The physics behind the process of collisional focusing is lucidly summarized in several references^9^, ^107^, ^120^ as well as in the original publication of its discovery.^119^ Briefly, at gas densities that are high (low milliTorr to Torr) relative to the vacuum requirements for mass filtering (<10^−5^ Torr), the collisions of the ions with the background gas molecules deplete their RF‐generated radial kinetic energy toward the rods and their gas flow generated axial kinetic energy along the ion path axis (axial and radial directions indicated in Figures 5B and 6B). The loss of their kinetic energy allows them to settle in the pseudopotential well on the centerline of the rods or lenses where the field is essentially zero. As shown in the drawing and simulation in Figures 9A and 9B this results in the ions concentrating in the centerline of the device allowing them to be focused through small gas‐restricting apertures at the exit. The ion‐to‐gas ratio is thereby increased. Several differentially pumped stages can be used to gradually reduce the pressure from the Torr to milliTorr regime with minimal ion losses prior to entering high vacuum for mass analysis and detection at ~10^−5^ Torr for quadrupoles.

The reduction in the ion's axial kinetic energy provided an additional benefit. It resulted in a monoenergetic beam of ions ideal for injection into a mass analyzer where variations in ion energies within a population of ions has a detrimental effect on mass resolution. This process of collisional cooling and focusing improved both sensitivity and mass resolution for a quadrupole mass analyzer at the same time which is an unusual outcome. It provided a path forward for continued sensitivity improvements by enabling greater ion‐containing gas loads to enter the vacuum system pumped by larger turbomolecular pumps (>500 L/s pumping speed, for example) without incurring excessive scattering losses.

Collisional focusing also made possible the design of miniaturized API‐MS systems having smaller vacuum pumps and lower ion‐containing gas loads. Gas passing through smaller atmosphere‐to‐vacuum apertures, similar to those used in the 1980s and early 1990s (25–250‐μm diameters), could be designed to be evacuated with scaled‐down turbomolecular pumps (10 L/s, for example) provided collisional focusing elements were used to pass the ion beam through successively smaller gas‐restricting lenses. The sampling efficiencies of some of these miniaturized benchtop and portable instruments could thereby exceed those of systems built in the 1980s and early 1990s having similar aperture dimensions but used large expensive vacuum systems such as those based on cryogenic pumping (see Figure 8). Sensitivity will be compromised as the gas load from the ion source is restricted but the application of miniaturized systems is for situations where size, portability, and cost reduction are more important than sensitivity and the lowest possible LOQ.

The collisional focusing principle was also applied, and near universally used today, in collision cells by raising their operating pressure into the low‐milliTorr range in cells that are enclosed with small entrance and exit apertures.^93^ As depicted in Figure 9C, ion‐scattering losses of the product ions are reduced by the collisional focusing effect. In addition, the collisions rob the product ions of their axial kinetic energy. The addition of an axial voltage gradient along the length of the cell^122^, ^123^ (one method of doing this is depicted in Figure 9D) prevents the ions from stopping particularly when the ion beam is periodically interrupted during MRM switching. These advances improved the transmission of ions through collision cells, improved their transmission into the second mass analyzing quadrupole, and improved mass resolution by eliminating the ion energy spread between the different product ions that occurs during collision and fragmentation. This was implemented in the mid‐1990s as indicated in Figure 8 and improved ion transmission efficiency approximately 10‐fold over previous collision cell designs.

Providing all of the above‐mentioned benefits enabled designs for large, high sensitivity instruments, made possible small, miniaturized low‐cost instruments, improved on mass resolution, and increased MS/MS transmission efficiency. This places the discovery of collision focusing as a significant advancement in the field of mass spectrometry that was first tested and implemented on QqQ instruments.

In summary to this section on sensitivity and sampling efficiency and referring to the graph in Figure 8, in the time between the mid‐1970s and mid‐1990s the sensitivity of QqQ instruments equipped with API sources was increased approximately 100‐fold by applying greater vacuum pumping speeds and increasing the vacuum aperture area. The use of high‐speed cryogenic pumps was responsible for most of these advances. In the mid‐1990s, as a consequence of the discovery of collisional focusing, vacuum apertures were further increased and ion‐scattering losses avoided resulting in an additional ~36‐fold increase in the vacuum aperture area from then to 2020. This improved ion transport efficiency with greater gains obtained from ion sources where the ionization process is widely dispersed, such as with conventional LC sample introduction, compared to the highly localized ionization with NanoESI where this efficiency has been high since its inception in the mid‐1990s. Greater gains in ionization efficiency were also achieved with conventional LC flows versus NanoESI where there was more room for improvement by increasing desolvation rates. Including the ~10‐fold improvement to ion transport through collision cells, sensitivity has increased by one‐million‐fold from the mid‐1970s to present for conventional API where ion production is widely distributed. It has increased by ~10,000‐fold between the mid‐1990s and today. For NanoESI, which was introduced in the mid‐1990s, the sensitivity has increased ~100‐fold because the sampling efficiency has always been very high leaving less room for improvement.

## 4 ION ACCOUNTING

**FIGURE 10:**
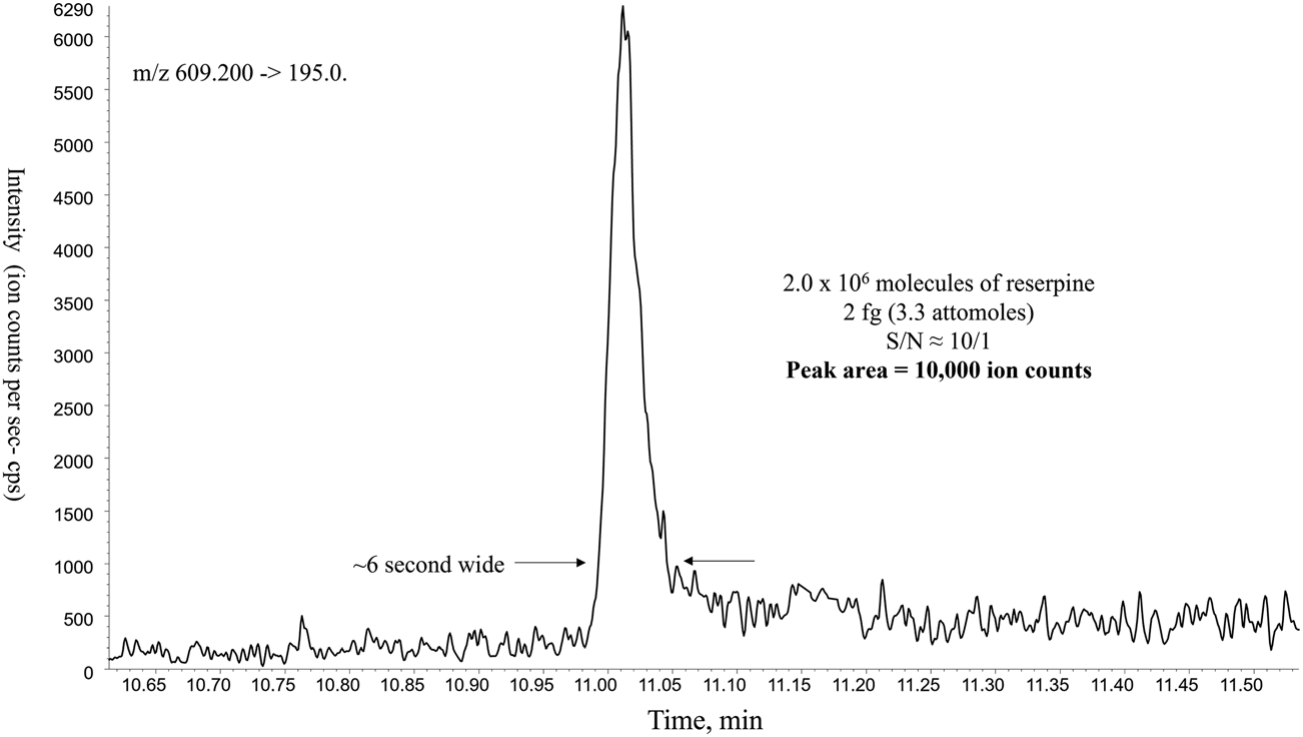
MRM ion current trace at the limit of quantitation for reserpine monitoring *m/z* 609 ➔ 195. Sample introduction was through a 2.1 mm × 3 cm HPLC column with 50:50 methanol/water mobile phase at 500 μL/min. The QqQ used was the same as in Figure 3 and the most recent point in the graphs of Figures 2 and 8. Sample amount injected was 2 fg (3.3 attomoles) corresponding to 2 × 10^6^ molecules producing a peak area of 10,000 ion counts. Baseline chemical noise at ~500 cps (counts per second). This means that from 200 molecules injected (330 yoctomoles) one ion would arrive at the detector. Adapted from Covey.^7^

An accounting of where all the ions go can provide the basis for an estimate of the feasibility and magnitude of future sensitivity improvements and from where they may come. Figure 10 shows the limit of quantitation for the reference compound reserpine that has been used over the years to assess the sampling efficiency of various instruments over time.^90^, ^91^, ^92^ Figure 3 indicates a 5.25% sampling efficiency under these conditions which means that 1.0 × 10^5^ ions arrive at the entrance to the first quadrupole mass analyzer from the 2 × 10^6^ molecules injected. With approximately 40% of the ion beam passing through a quadrupole mass filter at unit resolution, which results in a transmission of 16% under MS/MS conditions, 16,000 ions would be expected to enter the detector. Considering the losses incurred from converting the precursor ions into multiple product ions in the collision cell, the 10,000 ions being registered at the detector indicted by the peak area measurement in Figure 10 is in good agreement with the 16,000 ions calculated to arrive at the detector from the sampling efficiency measurement and mass filter losses. Extrapolating from this data, one ion will reach the detector from 200 molecules (330 yoctomoles) injected into the ion source. What lies beyond yoctomoles is nomormoles!

Future prospects for sensitivity increase from those areas where the losses are the greatest, i.e. sampling efficiency, mass filtering, and CAD fragmentation, are considered separately below as well as the possibility of improving detection limits by lowering chemical noise. The baseline in Figure 10 indicates at least 500 ions (165 zeptomoles) would be required to observe the signal above the chemical noise in this case.

### 4.1 CAD fragmentation

The transmission of product ions is highly efficient due to collisional focusing as described earlier. The percentage of the molecular ion current that is distributed into multiple product ions during CAD fragmentation is a function of a molecule's structure so cannot be altered and is highly variable. The only way to improve this is through derivatization which will not necessarily result in high selectivity if the product ion is not specific to the structure of interest. In addition, derivatization adds complications to analytical methods. Universally useful improvements to this component of ion losses are not encouraging although in certain cases derivatization can help.

### 4.2 Mass filtering

A large improvement to the transmission through quadrupole mass filters operated with RF and DC potentials is unlikely as it is determined by the limitations presented in the Mathieu equations. One advantage of quadrupoles operating in the SIM or MRM mode is that the duty cycle loss is very low when monitoring only a few transitions. Losses that occur from segmenting the continuous ion beam produced by ESI or APCI into pulses, as required with TOF and trap mass analyzers, do not occur.

The ion transmission characteristics of hybrid quadrupole TOFs and traps have typically been on the order of 5% or less of a QqQ in MRM mode which explains the enduring domination of the QqQ in the quantitation field. To a large degree this is the result of the duty cycle mismatch between the ion source and mass analyzer as mentioned above plus other areas of losses within these analyzers. Great strides to the improvement in the duty cycle of TOFs and traps is the topic of active research and is beginning to converge on the efficiency of the QqQ. For example, using a linear ion trap to eject slow‐moving high‐mass ions before fast‐moving low‐mass ions produces a scenario where all ions arrive in the TOF pulsing region at the same time. This approach reduces the mass‐dependent losses that occur during the pulsing of ions into the TOF analyzer.^124^

One option to reduce losses from mass filtering would be to abandon MS/MS and rely on high mass resolution alone for the selectivity required to reduce the noise component of the signal‐to‐noise equation. Eliminating the losses of the first quadrupole mass analyzer as well as the CAD fragmentation losses could bring the high‐resolution analyzers close to or better than the sensitivity of the QqQ. But without MS/MS one option for the quantitation of exact mass isobars would be lost requiring HPLC or some other form of chemically based pre‐separation. One convenient advantage over the QqQ is that the requirement to pre‐select conditions to transmit and fragment only the targeted ion of interest is no longer required with a TOF or trap because all product ions are collected with each pulse of them into the analyzer. Nevertheless, it is not apparent in general application that LOQs matching or significantly beyond today's QqQ in MRM mode can be expected soon with high‐resolution analyzers except in some selected cases when all aspects of the method align in their favor.

*Sampling efficiency* is the area from where gains remain to be had. With regards to the ionization efficiency component, for compounds that are poor ionizers the only apparent option for improvement is derivatization. In the case of electrospore compounds like reserpine their ionization efficiency is approaching 100% which means future gains can only come from improved atmosphere‐to‐vacuum transport and focusing of those ions in the gas expansion. At high flow rates it is unreasonable to expect more than a 10‐fold improvement because, as the 5.25% sampling efficiency suggests, a 10‐fold increase would then nearly match NanoESI which is close to the theoretical maximum. An end to improvements in electrospray mass spectrometry sensitivity are within sight as the limits are being approached. They have already been achieved with NanoESI.

In practice it is signal‐to‐noise (S/N) that counts and not just signal (sensitivity) so future improvements to LOQs beyond the ~10‐fold available from sensitivity improvements are possible. High chemical noise is endemic to API sources^2^ that comes from the sample matrix, contaminants in solvents, reagents, and environmental sources. Background ions form multiple clusters with water and solvent thereby occupying nearly every *m/z* channel under mass 1000 with several different species.^125^, ^126^ Developments in the area of chemical noise reduction are progressing using techniques that increase specificity by leveraging gas‐phase ion chemistry in reaction cells,^127^, ^128^ MS^3^ techniques, and higher mass resolution. Chemical noise reduction is one of the motives behind the ion mobility pre‐filters shown in the Histomap in Figure 1 and improvements to ion transmission through mobility analyzers could play a role in lowering LOQs. Capitalizing on the remaining improvements to sensitivity just discussed and further reductions in chemical noise it is conceivable that LOQs could be improved beyond today's benchmarks by 10–30‐fold. But the extraordinary gains for API QqQs achieved in the past 40 years will not be repeated in the future.

## 5 SENSITIVITY‐ENABLING APPLICATIONS. HIGH‐THROUGHPUT ANALYSIS

One obvious motivation to improve sensitivity over this time has been to lower limits of identification and quantitation but it has provided other dividends as well, more accurate mass measurements due to better ion statistics, and the enabling of additional prefilters such as mobility, to name but a few. One application not originally appreciated was the improvement to sample throughput. It improves throughput because the sample volumes required to achieve biologically relevant LOQs have been reduced proportional to the sensitivity gains which simplifies and generalizes sample preparation, enables the use of low‐volume high‐speed sample dispensers, reduces mass spectrometer contamination when subjected to high sample loads, and enables a means to control ionization suppression by applying large dilution factors to the sample. In the 1980s 1 mL of plasma, suitably extracted and concentrated, was required to achieve LOQs in the ng/mL or nM range. Today approximately 10^5^–10^6^ less sample is required to do that, i.e. low‐nL quantities, which can be directly injected without sample preparation provided sufficient dilution is used to manage ionization suppression.^7^, ^86^

The development of means to dispense small volumes of fluids in the picoliter to nanoliter range for various industrial, consumer, and biomedical research applications has been ongoing for the past 50 years.^129^ For example, the use of acoustic energy pulses to dispense low nanoliter volume droplets at speeds in excess of 1 Hz has emerged as a useful tool for managing large pharmaceutical compound libraries.^130^ However, the utility of such dispensers as a means for directly injecting biological fluids and assay buffers at high speed for mass spectrometry analysis has been hampered by a lack of sensitivity until recent years.

In the past decade a variety of high‐throughput, low‐volume dispensing techniques achieving 1–20 Hz sample injection rates have emerged in the MS field. Techniques like MALDI,^73^, ^131^ IR MALDESI,^132^, ^133^ and LAP MALDI^134^, ^135^ use lasers to dispense small sample amounts at high speed. In the case of MALDI the sample is introduced as a solid in a UV‐adsorbing matrix and the ionization is a form of rapid thermal desorption chemical ionization at low pressure. In the two other approaches the sample is introduced in liquid form at atmospheric pressure and droplets are ablated from the surface when the laser energy is absorbed. Molecules in the droplets are ionized via the ion evaporation mechanism as they are with ESI. Microfluidic‐based droplet segmenting devices,^136^ and the use of acoustic sound waves to dispense picoliter^84^ and nanoliter volume droplets^85^, ^86^ with ESI, are two additional low sample volume, high‐throughput technologies emerging.

Currently the above‐mentioned technologies are in various stages of development but all show promise having individual strengths and weaknesses. As of the date of this writing two have achieved commercialization status, MALDI and acoustically dispensed nanoliter volume droplets, i.e. acoustic ejection mass spectrometry (AEMS). ^86^ High‐throughput MALDI for drug discovery applications has been recently summarized and represents an approach with ionization of solid samples under vacuum conditions.^131^ The electrospray‐based approach of AEMS will be used here to illustrate the impact on high‐throughput mass spectrometry and the sensitivity gains that have been made with API QqQs, the theme of this review.

**FIGURE 11:**
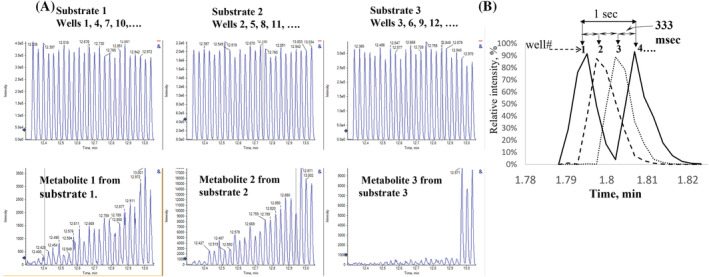
Chronograms generated using acoustic ejection mass spectrometry (AEMS) for three cytochrome P450 drug–drug interaction assays. Samples were in a 384‐well microtiter plate. The term chronogram is used because there is no chromatographic separation involved. A volume of 2.5 nL is acoustically ejected and captured by an Open Port Interface, without sample pre‐purification, at a rate of 3 samples per second. The open port interface captures, dilutes, and transports the acoustically generated drop in a flowing liquid stream into a classical ESI source typically used for LC/MS. (A), The top row of chronograms is the signal from three different parent drugs at high concentration (high nM) and the appearance of their metabolites at low concentrations (low nM) in the bottom row of traces. Spacing between each peak for a single compound is 1 s. Adjacent wells, injected at a rate of 333 ms per well, contain a different parent drug being tested in this assay. Three different drugs are being tested so that drug #1 will be in wells 1, 4, 7.., drug #2 in wells 2, 5, 8 …, and drug 3# in wells 3, 6, 9 …, etc. Coefficients of variance (CVs) obtained for this assay were 8%. The signal from 72 wells is shown. (B), Expansion of the chronogram shown in (A) over four injections to illustrate the multiplexing method that enables a throughput of 3 samples per second. The multiplexing method requires that a different compound with different MRM transitions is being assayed in adjacent wells. The peaks overlap but the signals are distinguished by the different MRM transitions from the different compounds. Figure reproduced with permission from Covey^7^ [Color figure can be viewed at wileyonlinelibrary.com]

The ability to dispense single digit nanoliter volume droplets accurately and reproducibly from microtiter plates at high speed with acoustic energy solves only a part of the problem. The droplets must either be charged so they will emit ions or transported in a flowing liquid stream at speeds comparable to the dispensing rate into an ESI source for ionization. One way to achieve the latter is using the Open Port Interface which has demonstrated the ability to keep nanoliter volume droplets segregated in a flowing liquid stream during transport into the ion source. As shown in Figure 11 single digit nanomolar LOQs can be achieved with favorable compounds by directly injecting 2.5 nL volumes of a cytochrome P450 drug–drug interaction assay with no sample preparation at speeds exceeding 1 Hz. This has enabled routine daily sample throughputs on the order of 70,000 per day^137^ of various pharmaceutical drug discovery HTS and ADME assays.^7^, ^138^, ^139^, ^140^, ^141^, ^142^, ^143^, ^144^, ^145^, ^146^

**FIGURE 12:**
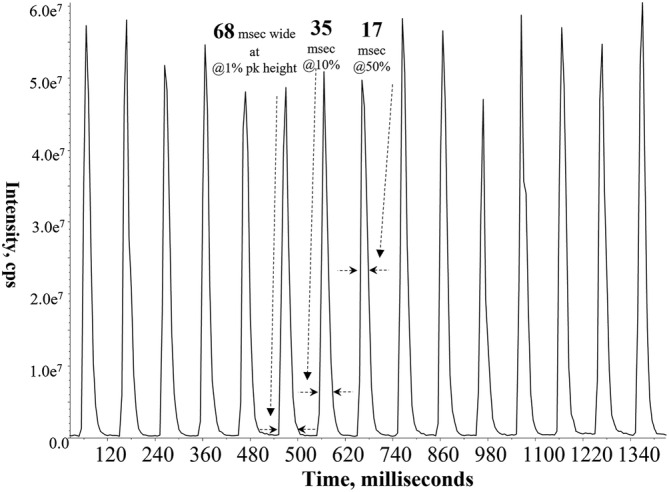
Chronogram using an open port interface modified to obtain maximum speed and with 5 nL droplet introduction using a fast pneumatically driven nanoliter dispenser originally designed for 3‐D printing of biological materials and matrices (http://www.3dispense.com/dispensing.html). Here it is operating at 10 sample dispenses per second. Based on the separation of the peaks at baseline this demonstrates the feasibility of HTMS speeds up to 15–20 Hz. Compound monitored by MRM (5 ms dwell time) was DEET with the transport flow rate 2.5 mL/min. Adapted from Liu et al^85^

The use of acoustic energy to accurately and reproducibly dispense nanoliter volume samples is limited to <10 samples per second as implemented to date.^86^, ^137^ The Open Port Interface, however, can transport samples into an ESI source faster demonstrating speeds approaching 20 Hz, as seen in Figure 12.^85^ The earlier mentioned techniques of LAP‐MALDI,^135^ IR‐MALDESI,^132^ and microfluidic droplet segmenting devices^136^ have shown similar speeds. If proven to be sustainable on a 20‐h basis, this translates to ~1.4 million sample measurements per day with a single mass spectrometer. In the 1980s the standard ritual upon entering an LC/MS testing laboratory then was to spray n’ pray in the desperate hope that one‐to‐ten LC/MS chromatograms could be completed that day before a rough pump seized, a high‐voltage discharge intervened, or other catastrophic system failures occurred. When the various technologies to couple MS and LC were emerging at that time, discussions regarding the possibility of tens to hundreds‐of‐thousands samples per day would have been viewed by most as the rantings of a mad man, no more possible than the concept of every individual in society being able to carry a computer in their pockets having the power of a large mainframe from that era.

## 6 CONCLUSIONS

Gains to the sensitivity of triple quadruple mass spectrometers equipped with atmospheric pressure ionization sources were largely driven by the needs of the biomedical research community and other scientific, industrial, and regulatory enterprises to achieve lower limits of quantitation and identification and to do so with increasingly small sample sizes. A one‐million‐fold improvement was incrementally achieved over the past 40 years by the various manufacturers of mass spectrometers improving efficiencies in several areas. The discovery of collisional focusing spawned the development of high‐pressure RF ion guides having a variety of different geometries which enabled effective differentially pumped designs to introduce greater ion‐containing gas loads to be transferred into vacuum with reduced ion‐scattering losses and improved the efficiency of their transfer through collision cells. Larger turbomolecular vacuum pumps were designed to provide the increased pumping speed that collisional focusing permitted. The collisional focusing effect was also used to reduce the size, cost, and portability of MS systems by enabling lower gas loads and smaller pumps to be used while maintaining sensitivity levels appropriate for applications where those metrics were more important than having the highest possible sensitivity. Significant sensitivity gains were achieved through a deeper understanding of the ionization process recognizing the importance of optimizing droplet desolvation conditions for improving the efficiency of electrospray ion production across the liquid inlet flow range used for HPLC.

These developments have brought the field to a point where the limits to improvements in sensitivity are in sight. Today only about 200 molecules are required for one ion to reach the detector and, in the case of NanoESI, only 20. This is in stark contrast to electron impact ionization under vacuum where one in every 10^3^–10^4^ molecules introduced exit the ion source as ions which is then followed by additional losses during mass filtering and ion path transmission.

Currently, with favorably ionizing compounds, a 70% sampling efficiency can be obtained with NanoESI and a 5% sampling efficiency for both ESI and APCI operating at sample introduction liquid flow rates typical of conventional LC. This leaves room for an additional sensitivity improvement of ~10‐fold for conventional LC/MS in the future. It is shown here that 2 × 10^6^ molecules (3 attomoles) of reserpine will deliver to the detector 10^4^ ions in MRM mode and produce a quantifiable signal with a S/N ~10:1. This suggests that chemical noise reduction through the use of ion mobility, high‐resolution mass analyzers, or gas‐phase ion chemistry reaction cells, along with capitalizing on the remaining sensitivity gains available, a 10–30‐fold reduction in LOQs could be achieved in the future provided excessive numbers of ions are not lost in the process used to improve selectivity. This would improve the current detection levels from low attomoles to zeptomoles for electrospore‐like compounds.

One consequence of this one‐million‐fold gain in sensitivity is sample volumes one‐million‐fold less than several decades ago can now be analyzed while maintaining biologically relevant LOQs. This has enabled the use of a variety of new high‐speed low‐volume dispensing technologies to dramatically increase sample throughput. Shown here is that 70,000 samples/day is routinely sustainable but some new emerging technologies indicate the possibility of throughputs ten‐fold greater. This is a concept unthinkable 40 years ago when one‐to‐ten successful LC/MS analyses per day was considered an achievement.

It is thought provoking to consider the implications of this beyond the analysis of more samples per day, as compelling the prospect of increased throughputs presents. It now appears feasible to analyze 20 samples, equivalent to an entire calibration curve, in the blink of an eye. This suggests that real‐time feedback for self‐checking and troubleshooting both the instrumentation status and the entire analytical method could be implemented. One function could be the detection of excessive ionization suppression in samples of unexpected composition and the correction of the measurement in near real‐time by the immediate repeat of the analysis using a higher dilution ratio.

Since the sensitivity gains of the past four decades can no longer be repeated in their magnitude, the future now lies in taking full advantage of these past labors to expand the capabilities of atmospheric pressure ionization on triple quadrupole and other types of mass spectrometers to address new areas of chemical analysis. Low sample volume, high‐throughput technologies and applications are examples of how the sensitivity gains can be leveraged to open new areas of application. There are many others waiting to be discovered, championed, and nurtured into a form where they can be successfully deployed for routine use. Standardized, cumbersome, and often misappropriated industrial processes increasingly used to control the commercialization of new technologies may be the biggest threat to the deployment of this type of innovation. However, I have no doubt that today's cohort of bright young scientists and engineers will find ways, by hook or by crook, around these institutional barriers and advance the field of mass spectrometry, in one fashion or another, an additional million‐fold in the coming decades. “To you from failing hands we throw the torch; be yours to hold it high.” From in “Flanders Fields“ by Lieutenant‐Colonel John McCrae of the Canadian Expeditionary Force after the Second Battle of Ypres, May 1915.

### PEER REVIEW

The peer review history for this article is available at https://publons.com/publon/10.1002/rcm.9354.

## Data Availability

The data that support the findings of this study are available from the corresponding author upon reasonable request.
